# Characterization of DNA Methylomic Signatures in Induced Pluripotent Stem Cells During Neuronal Differentiation

**DOI:** 10.3389/fcell.2021.647981

**Published:** 2021-07-01

**Authors:** Jennifer Imm, Ehsan Pishva, Muhammadd Ali, Talitha L. Kerrigan, Aaron Jeffries, Joe Burrage, Enrico Glaab, Emma L. Cope, Kimberley M. Jones, Nicholas D. Allen, Katie Lunnon

**Affiliations:** ^1^Institute of Biomedical and Clinical Sciences, University of Exeter Medical School, University of Exeter, Exeter, United Kingdom; ^2^Department of Psychiatry and Neuropsychology, School for Mental Health and Neuroscience, Maastricht University, Maastricht, Netherlands; ^3^Biomedical Data Science Group, Luxembourg Centre for System Biomedicine, University of Luxembourg, Belvaux, Luxembourg; ^4^School of Biosciences, Cardiff University, Cardiff, United Kingdom

**Keywords:** aging, DNA methylation, EPIC array, epigenetics, epigenome-wide association study, induced pluripotent stem cells, neuronal differentiation, trajectory inference

## Abstract

In development, differentiation from a pluripotent state results in global epigenetic changes, although the extent to which this occurs in induced pluripotent stem cell-based neuronal models has not been extensively characterized. In the present study, induced pluripotent stem cell colonies (33Qn1 line) were differentiated and collected at four time-points, with DNA methylation assessed using the Illumina Infinium Human Methylation EPIC BeadChip array. Dynamic changes in DNA methylation occurring during differentiation were investigated using a data-driven trajectory inference method. We identified a large number of Bonferroni-significant loci that showed progressive alterations in DNA methylation during neuronal differentiation. A gene–gene interaction network analysis identified 60 densely connected genes that were influential in the differentiation of neurons, with *STAT3* being the gene with the highest connectivity.

## Introduction

Neuronal development is a complex and protracted process that begins early in gestation with the differentiation of stem cells into neuronal progenitors (Stiles and Jernigan, 2010). Both gene expression and environmental stimuli are known to be very important regulators of this process, and disruptions to either of these can ultimately affect brain development (Stiles, 2008). One process through which gene expression can be controlled is via epigenetic mechanisms, the most widely studied of which is DNA methylation (Henikoff and Matzke, 1997; Lande-Diner et al., 2007). There is considerable literature that shows that DNA methylation changes occur throughout the life course and in every tissue and cell type (Kitamura et al., 2007; Illingworth et al., 2008; Murgatroyd and Spengler, 2011). These changes begin very early in the brain, with dynamic DNA methylation changes reported across fetal development (Spiers et al., 2015), and further changes throughout the life course as we age (Horvath and Raj, 2018).

As neuronal development is such a critical process, it is important to understand the epigenetic changes that drive cell specification and differentiation in the brain using the most appropriate model system that can recapitulate the dynamic changes occurring in this process. Owing to advances in genomic technology, epigenome-wide association studies (EWAS) of DNA methylation have been undertaken to study the methylomic trajectories of fetal brain development (Spiers et al., 2015; Li et al., 2020). However, while these studies have provided considerable insight into the epigenomic landscape of neuronal development, there are caveats to this approach. For example postmortem brain tissue was used, with different samples analyzed at different stages of development, which thus have different genetic backgrounds, which is known to impact the epigenome (Gutierrez-Arcelus et al., 2013).

One promising avenue for longitudinal modeling of neuronal development is through the use of induced pluripotent stem cell (iPSC)-derived neuronal cells, as iPSCs are derived from human tissue, can be monitored over time, and, in theory, can be transformed into any cell type in the body while having the same genetic background (Imm et al., 2017). Despite iPSC-derived neurons being functionally mature, displaying mature electrophysiological features, including spontaneous electrical activity, regenerative induced action potential train activity, and hyperpolarized resting membrane potentials (Telezhkin et al., 2016), they do not retain their age-related transcriptomic or epigenomic profiles (Mertens et al., 2015). This lack of maturity makes them a good candidate for studying neuronal development.

To our knowledge, no studies have yet longitudinally profiled DNA methylation patterns and the epigenetic “age” of iPSC-derived neurons during differentiation and maturation. This is an important area to investigate as it will provide valuable information on the development of neurons, which could later be compared to iPSC-derived neurons harboring mutations known to affect neurological function. In this study, we have differentiated iPSCs into cortical neurons and assessed their DNA methylation profile at different time points during differentiation and maturation to identify DNA methylomic trajectories of neuronal differentiation.

## Materials and Methods

### iPSC Culture and Neuronal Differentiation

This study was performed using one extensively characterized feeder-free human iPSC line (33Qn1), originally derived from human fibroblasts by transfection of episomal plasmid vectors expressing the six transcription factors Oct4, Sox2, Klf4, cMyc, Nanog, and Lin28 (The Hd iPsc Consortium, 2012). Differentiation was achieved using the SCM1/2 protocol outlined in Telezhkin et al. (2016). Briefly, iPSCs were maintained on vitronectin-coated plates in an Essential 8 flex medium, passaged using dispase according to the manufacturer’s instructions (Stem Cell Technologies, Vancouver, Canada), and were collected 4 days after initial plating for DNA extraction (Day 0—iPSCs). Neuronal differentiation was started at approximately 70% confluency. Differentiation into neuronal precursors was achieved using SLI media (Advanced DMEM:F12 (with Glutamax); 1% Penicillin/Streptomycin; all from Life Technologies, California, United States); 10 μM SB431542 (Abcam, Cambridge, Cambs., United Kingdom); 1 μM LDN 193189 (Stemgent, Cambridge, MA, United States); 1.5 μM IWR1 (Tocris Bioscience, Abingdon, Oxon., United Kingdom); and 2% NeuroBrew-21 without RA (Miltenyi Biotec: Bisley, Surry, United Kingdom) for the first 8 days followed by LI media for another 8 days (Advanced DMEM:F12, 2 mM L-glutamine, 1% Penicillin/Streptomycin, 200 nM LDN 193189, 1.5 μM IWR1, and 2% NeuroBrew-21 without RA), after which neuronal precursor cells (NPCs) were collected for DNA extraction (Day 16—NPCs). The remaining neuronal precursors were then terminally differentiated and matured as described previously (Telezhkin et al., 2016) using the sequential addition of the SCM1 for 7 days {SCM1 contained Advanced DMEM:F12 (with Glutamax); 1% penicillin/streptomycin; 2% NeuroBrew21 (Miltenyi Biotec); 2 μM PD0332991 (Selleckchem); 10 μM DAPT (Sigma-Aldrich); 0.6 mM CaCl_2_ [to give 1.8 mM total CaCl_2_ in final complete medium (Sigma-Aldrich); 200 μM ascorbic acid (Sigma-Aldrich); 10 ng/mL BDNF (Miltenyi Biotec); 1 μM LM22A4 (Tocris Bioscience); 10 μM Forskolin (FSK, Tocris Bioscience); 3 μM CHIR 99021 (Tocris Bioscience); and 300 μM GABA (Tocris Bioscience)]} and then SCM2 for the remainder of the maturation period for a further 37 and 58 days {SCM2 contained 1:1 Advanced DMEM/F12 (with Glutamax): Neurobasal A (Life Technologies); 1% penicillin/streptomycin (Life Technologies); 2% NeuroBrew21 with RA (Miltenyi Biotec); 2 μM PD0332991 (Selleckchem); 3 μM CHIR 99021 (Tocris Bioscience); 0.3 mM CaCl_2_ [to give 1.8 mM total CaCl_2_ in the final complete medium (Sigma-Aldrich); 200 μM ascorbic acid (Sigma-Aldrich); 10 ng/mL BDNF (Miltenyi Biotec)]}. At these time points, the cells were collected for DNA extraction (Days 37 and 58—mature neurons). At each time point, cells were collected separately from four wells, representing four technical replicates. All collected cells were washed with PBS, pelleted down, frozen, and stored at –80°C.

### Genome-Wide Quantification of DNA Methylation

DNA was extracted from the 16 cell pellets using a standard phenol chloroform protocol. Subsequently, 500 ng of genomic DNA was sodium bisulfite converted using the Zymo EZ 96 DNA methylation kit (Zymo Research) according to the manufacturer’s instructions. Samples were profiled using the Illumina Infinium Human Methylation EPIC BeadChip array (Illumina) and the Illumina HiScan System (Ilumina).

All data analysis was performed in R version 3.6.1 (Eggshell Igloo). The methylumi package (Davis et al., 2017) was used to extract signal intensities for each CpG probe and perform initial QC, with data normalization and preprocessing using the WateRmelon package (Pidsley et al., 2013). Additional QC checks were performed using the “p-filter” function within the methylumi package, assessing bisulfite conversion efficiency, and the median methylated and unmethylated sample intensities as previously described (Smith et al., 2019). Two iPSC samples failed the p-filter checks due to low median (un)methylated sample intensities. As a result, they were removed from the study. For the remaining 14 samples, the data were normalized with the *dasen* function from the wateRmelon package (Pidsley et al., 2013). Prior to any analyses, probes with common (>5% minor allele frequency [MAF]) single nucleotide polymorphisms (SNPs) within 10 bp of the single base extension and probes with sequences previously identified as potentially hybridizing to multiple genomic loci were excluded (McCartney et al., 2016), resulting in a final dataset of 837,018 probes.

### Epigenetic Age Calculation

In this study, we used two epigenetic age calculators: first, the pan-tissue DNA methylation age estimator (Horvath et al., 2018), which predicts chronological age based on the DNA methylation levels of 353 CpGs that were identified in human adult tissues, and second, a recently developed epigenetic clock using DNA methylation data from fetal brain tissue, based on 107 CpGs (Steg et al., 2020). The coefficients and intercept for both the Horvath and Steg age calculators were downloaded and were applied using the *agep* function of the wateRmelon package (Pidsley et al., 2013). The epigenetic ages calculated by the Horvath clock were then converted from years to days post-conception to allow comparisons to be made. To test for differences in predicted epigenetic age between cell stages for each clock, we used an ANOVA followed by Tukey’s honest significant difference (HSD) test to allow for multiple comparisons.

### Probe Filtering and Dimensionality Reduction

Median absolute deviation (MAD) was computed as a robust measure of variability for each CpG site across the four cell stages, with the upper fifth percentile value used as a cutoff to determine the most variably methylated loci (41,851 loci). Principal component analysis (PCA) without scaling the probes by their variance was then applied to obtain a lower-dimensional feature subspace, representing the information explaining most of the variance in the dataset.

### Pseudotime Trajectory Analysis

A pseudotime trajectory through the cell stages was inferred and plotted using the *infer_trajectory* and *draw_trajectory_plot* functions in the SCORPIUS package (version 1.0.7), respectively (Cannoodt et al., 2016). The first two principal components of the DNA methylation data were subjected as the coordinate of the samples to the *infer_trajectory* function, which performs k-means clustering, calculates the distance matrix between cluster centers and finds the shortest path connecting all cluster centers using a custom distance function, and finally fits a curve to the given data using principal curves (Cannoodt et al., 2016).

Next, to identify the loci with the largest contribution to the trajectory inference, we regressed each CpG site’s methylation values on the pseudotime variable that had been inferred by trajectory analysis after rounding off the pseudotime values to two decimal places. We used a general additive model (GAM), which allowed the detection of non-linear methylation patterns throughout neuronal differentiation. The loci that remained significant after Bonferroni correction for 41,851 tests were considered as robust markers of neuronal differentiation and subjected to further downstream analyses.

### Gene Ontology, Pathway, and Genomic Enrichment Analyses

We performed Gene Ontology (GO) pathway analysis using the missMethyl R package (Phipson et al., 2016), which adjusts for the uneven number of CpGs per gene on the Illumina Infinium Human Methylation EPIC BeadChip array. Pathways are reported if they were false discovery rate (FDR) significant. To test for an enrichment of DMPs in specific genomic features (i.e., CpG islands, shelves, shores, and non-CpG island regions) and genomic regions related to transcription (TSS1500, TSS200, 5′UTR, 3′UTR, first exon, and gene body), we annotated all DMPs based on their Illumina annotation and performed a two-sided Fisher’s exact test.

### Gene–Gene Interaction Network Analysis

We used MetaCore (Clarivate Analytics) to obtain a set of directed functional regulatory interactions between the unique genes annotated to the CpG sites with the largest contribution to the trajectory inference. The MetaCore database contains a compilation of manually curated and experimentally validated directed gene–gene interactions based on existing literature. Its high level of manual curation ensures the creation of highly confident interaction network maps. The network reconstruction was restricted to interactions reported in humans from the categories “transcriptional regulation,” “influence on expression,” “co-regulation of transcription,” and “regulation” with the interaction type (i.e., activation or inhibition) provided when available. Subsequently, the R package igraph (version 1.2.6) (Csardi and Nepusz, 2006) was used to extract the strongly connected component (SCC) from the network obtained through Metacore. The *network analyzer* tool from Cytoscape (version 3.4.0) (Shannon et al., 2003) was used to conduct a network topological analysis in order to identify key genes with high centrality and connectivity in the network.

### Gene Expression Data

We utilized the iPSC transcriptomics resource generated by Burke et al. (2020) to explore the patterns of gene expression throughout the differentiation for genes identified in our gene--gene interaction network analysis. Their online tool^1^ contains RNA-sequencing data generated in 5 iPSC donor and 13 subclonal lines over a range of different conditions and timepoints postdifferentiation. For the purposes of quantifying gene expression changes for all genes identified in our gene-gene interaction network analysis we have extracted from their database the direction of change and FDR-adjusted *P* value (*Q*-value) over time for all samples in their study. We also downloaded directly from the website plots for selected genes of interest we identified.

## Results

### iPSC-Derived Neurons Have a Fetal Epigenome

**FIGURE 1:**
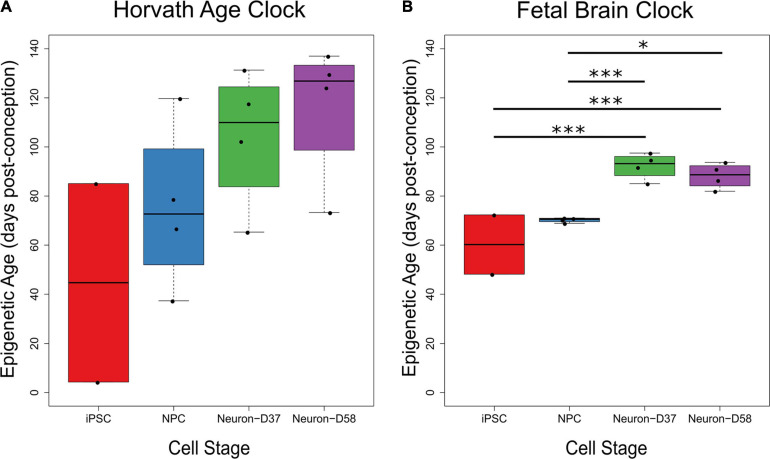
The predicted biological ages of iPSC-derived neurons through differentiation using two different clocks. As two iPSC samples did not pass the quality control checks, there are only two samples in the iPSC group on each graph. **(A)** The estimated epigenetic age (Y-axis) of the four cellular stages (X-axis) increased throughout differentiation using the epigenetic age clock created by Horvath et al. (2018), although there were no significant differences between cellular stages. **(B)** The estimated epigenetic age of iPSCs and NPCs were significantly lower than the mature Days 37 and 58 neurons using the fetal brain epigenetic age clock created by Steg et al. (2020), but with no difference between iPSCs and NPCs or between Days 37 and 58 neurons. The age of each sample is given in days post-conception. Key: ^∗^*P* < 0.05, ^∗∗∗^*P* < 0.005.

One concern when using iPSC-derived neurons to study diseases, particularly age-related neurodegenerative disorders, is the biological age of the neurons. To address this, we used two different epigenetic age calculators to calculate the epigenetic age of the samples, including the latest iteration of the Horvath age calculator (Horvath et al., 2018) and a new fetal brain clock developed by Steg et al. (2020). The Horvath calculator predicted an increase in epigenetic age throughout differentiation and maturation, although these increases were not significant (Figure 1A). However, the samples were all predicted to be fetal (i.e., <280 days post-conception), with the highest predicted epigenetic age being 137 days post-conception. The fetal brain clock also predicted all samples to be fetal, with the samples ranging from 48 to 97 days post-conception (Figure 1B). The fetal brain clock did not predict a completely linear increase in epigenetic age with differentiation status. There was a significant increase in predicted epigenetic age for the mature neuron stages compared to both the iPSCs and NPCs (iPSC v Day 37 neuron: *P* = 1.36 × 10^–3^; iPSC v Day 58 neuron: *P* = 3.57 × 10^–3^; NPC v Day 37 neuron: *P* = 4.72 × 10^–3^; NPC v Day 58 neuron: *P* = 0.0169), but with no difference between the iPSCs and NPCs, nor the Days 37 and 58 neurons.

### Cell Trajectory Modeling Highlights Methylation Patterns During Differentiation

**FIGURE 2:**
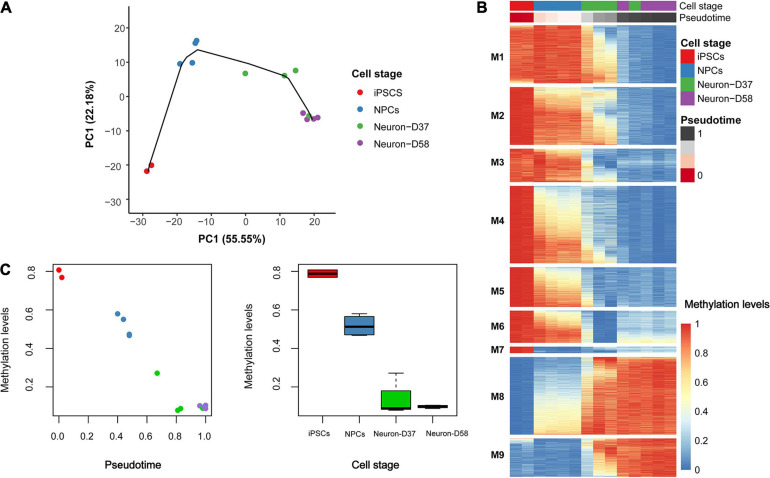
Trajectory inference modeling identifies a signature of 6,843 probes that distinguish cell stage. **(A)** To create the trajectory model dimensionality reduction was first performed, using principal component analysis (PCA), followed by estimating pseudo-time to model the lineage trajectory. The different samples grouped together based on the first two principal components (PCs). **(B)** Using the pseudo-time estimation, a generalized additive model (GAM) was used to determine which of the 6,843 probes were becoming hypomethylated (blue) or hypermethylated (red) over time. The patterns of hypomethylation and hypermethylation were grouped into nine modules (M1-9) that could distinguish the different cell stages. **(C)** The DNA methylation patterns occurring at the most significant probe (cg00908292) throughout differentiation. Left: plot of methylation beta-value (Y-axis) against pseudo-time (X-axis) and right: plot of methylation beta-value (Y-axis) against cellular stage (X-axis).

In order to further explore how DNA methylation levels change throughout neuronal differentiation and maturation, we generated a cellular lineage trajectory signature to identify groups of loci that become progressively hyper- or hypomethylated throughout differentiation. First, we reduced the dimensionality of the dataset containing the most variable CpG probes (41,851 loci) to 14 principal components (PCs), of which the first 2 explained ∼78% of the variation in that dataset. These two PCs were used as the coordinates for samples to cluster them according to the stage of differentiation/maturation (Figure 2A). Samples within each cellular stage clustered together, with the exception of one Day 37 neuron sample, which clustered with the Day 58 neuron samples. This could indicate that this sample had aged quicker than the others in the same group; however, the “epigenetic age” of this sample corresponded to the second youngest of the four Day 37 neuronal samples when using the Steg fetal brain clock. To ensure that this sample was not a general outlier, we clustered all 14 samples based on the Euclidean distance (prior to the trajectory inference analysis). This highlighted that this Day 37 sample was not an outlier in general and clustered together with the Day 58 samples (Supplementary Figure 1).

Using the pseudotime generated in the trajectory analysis as a predictor, a GAM was fitted to the 41,851 probes in order to identify the loci that contributed most to the trajectory inference model. In total, we identified 6,843 of the 41,851 loci that showed Bonferroni significant variation in methylation across the cell stages (Figure 2B), which we termed the “epigenetic trajectory signature.” Full details on these loci, including the change in methylation, genomic coordinates, and gene annotation, can be found in Supplementary Table 1. The Bonferroni significant loci were then grouped into nine modules according to their pattern of methylation changes across differentiation. Interestingly, the probes in module 7 (M7) seem to particularly characterize the iPSC stage, as they undergo an average decrease in methylation of 59% between the iPSC and NPC cell stages (Supplementary Table 2).

The largest change in DNA methylation occurring through differentiation occurs at the cg00908292 probe, which is intergenic and located closest to the *CCR7* gene (Bonferroni corrected *P* = 4.36 × 10^–9^). This locus is hypermethylated in iPSCs, becoming hemimethylated in NPCs, before becoming progressively demethylated over time and being largely unmethylated in the terminally differentiated mature neurons (Figure 2C).

### Pathway Analysis of Loci Contributing to the Epigenetic Trajectory Signature Implicates Neuronal Pathways

We took the 6,843 loci that comprised the epigenetic trajectory signature and used GO enrichment analysis to identify the most significant pathways that were changing throughout neuronal differentiation. The epigenetic trajectory signature resided in genes that featured in specific pathways relating to “neuron projection morphogenesis,” “cell growth,” and “movement of cell or subcellular component” (Supplementary Table 3 and Supplementary Figure 2).

### Enrichment of Loci in the Epigenetic Trajectory Signature in Specific Genomic Features and Regions

Next, we investigated whether loci comprising the epigenetic trajectory signature resided in specific genomic regions. Overall, we saw an enrichment of these probes in the gene body (odds ratio [*OR*] = 1.07, *P* = 0.021) and the 3′ untranslated region (3′UTR) (*OR* = 1.20, *P* = 0.048) (Supplementary Table 4 and Supplementary Figure 3). Interestingly, when we examined the enrichment of hypomethylated (*N* = 4,954) and hypermethylated (*N* = 1,889) loci independently, we observed a difference in their genomic location; hypomethylated loci were underrepresented within both 1,500 and 200 bp of the transcription start site (TSS1500: *OR* = 0.73, *P* = 2.33 × 10^–8^; TSS200: *OR* = 0.72, *P* = 4.80 × 10^–5^) and enriched in the gene body (*OR* = 1.20, *P* = 1.38 × 10^–9^), while hypermethylated loci were enriched in the TSS1500 (*OR* = 1.48, *P* = 1.39 × 10^–7^), TSS200 (*OR* = 1.99, *P* = 1.22 × 10^–12^), and 3′UTR (*OR* = 1.93, *P* = 2.85 × 10^–6^) and underrepresented in the gene body (*OR* = 0.77, *P* = 1.37 × 10^–7^). When we investigated the enrichment of loci within specific genomic features related to the CG content, we observed a significant enrichment of the loci comprising the epigenetic trajectory signature in CpG island (CGI) shelves (*OR* = 1.17, *P* = 5.09 × 10^–3^) and shores (*OR* = 1.22, *P* = 1.67 × 10^–8^) (Supplementary Table 4 and Supplementary Figure 3). Hypomethylated probes were significantly underrepresented in CGIs (*OR* = 0.26, *P* = 6.74 × 10–76) and shores (*OR* = 0.74, *P* = 9.65 × 10^–12^) and enriched in shelves (*OR* = 1.18, *P* = 0.011), while hypermethylated probes were significantly enriched in CGIs (*OR* = 3.82, *P* = 3.08 × 10^–103^) and shores (*OR* = 2.73, *P* = 6.23 × 10^–78^).

### Transcriptional Regulation Is a Highly Interconnected Process Throughout Differentiation

**FIGURE 3:**
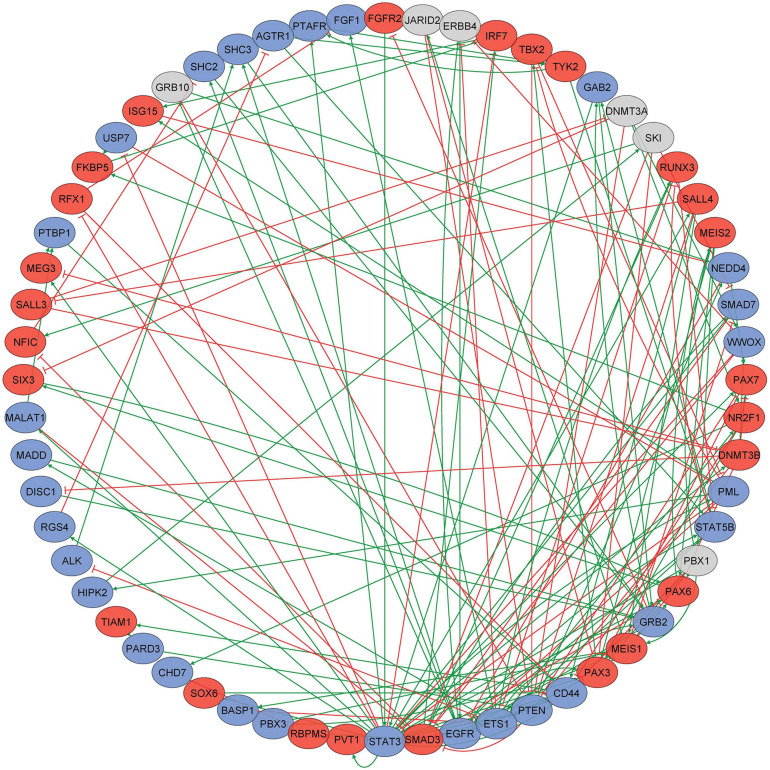
A subnetwork of 60 genes constituting the strongly connected component (SCC) in the gene–gene interaction network. Directed gene–gene interaction network was constructed for 2,659 unique genes that were annotated to the 6,843 loci comprising the epigenetic trajectory signature. The prior knowledge network (PKN) obtained from Metacore contained 398 genes and 622 interactions. Only one strongly connected component (SCC) in this network, comprised of 60 genes and 158 interactions between them, was identified; blue nodes indicate genes becoming progressively hypomethylated, red nodes indicate genes becoming progressively hypermethylated, and gray ovals indicate genes that have more than one probe annotated to them that have different patterns of methylation change.

To explore the connectivity between key genes that display progressive DNA methylomic changes through differentiation, we performed gene–gene interaction analyses on the 2,659 unique genes that were annotated (Illumina [UCSC] annotation) to the 6,843 loci comprising the epigenetic trajectory signature. The prior knowledge network (PKN) obtained from MetaCore contained 398 genes and 622 interactions. Only one strongly connected component (SCC) existed in this network (i.e., there is only one subnetwork in which every gene can be reached through any other gene in the same subnetwork), comprised of 60 genes and 158 interactions between them (Supplementary Table 5 and Figure 3). The conducted topological network analysis highlighted the key genes in this SCC with outstanding topological characteristics, including out-degree (set of target genes it regulates), in-degree (set of upstream regulating genes), betweenness centrality (most influential genes based on their shortest paths to other genes in the network), and clustering coefficient (a measure of gene tendency to cluster with other genes in the network). *STAT3* was the gene with the highest connectivity (Neighborhood Connectivity = 5.42, Clustering Coefficient = 0.045) in the SCC, according to its in-degree (7) and out-degree (20), suggesting that it may play a key regulatory role in the subnetwork. Previously, alterations in STAT3 signaling have already been observed to be associated with age-related changes in different cell types (Chazaud and Mouchiroud, 2014; O’Brown et al., 2015).

In order to add additional meaning to the methylation changes we see throughout differentiation, we assessed the gene expression changes of our 60 genes comprising the SCC using the online tool created by Burke et al. (2020) available at http://stemcell.libd.org/scb/. In total, 53 of the 60 genes in the SCC showed FDR significant gene expression changes in the Burke dataset (Supplementary Table 6 and Supplementary Figure 4). Included in these 53 genes are the epigenetic modulators *DNMT3B* and *DNMT3A* (Supplementary Figure 5) as well as loci known to be important for neuronal differentiation such as *PAX6* (Simpson and Price, 2002) *and STAT3* (Yadav et al., 2005; Snyder et al., 2011).

## Discussion

In this study, we quantified genome-wide DNA methylation in iPSCs and throughout their differentiation and maturation into cortical neurons. We have shown that iPSCs, NPCs, and post-terminally differentiated neurons have an immature epigenomic profile according to both the Horvath epigenetic age calculator and the Steg fetal brain clock. While the Horvath epigenetic age calculator has been shown to accurately predict the epigenetic age of brain samples (Horvath et al., 2018), it has been reported to be inaccurate in juvenile samples, presumably because DNA methylation changes are more dynamic in children (McEwen et al., 2019). For this reason, we also used the Steg fetal brain clock as this was trained using human fetal brain samples (Steg et al., 2020). Both methods predicted that the iPSC-derived neurons are epigenetically immature, with the Steg fetal clock showing significant differences between cellular stages. Interestingly, although there was a significant increase in the predicted epigenetic age using the Steg fetal clock between the iPSCs or NPCs and the Days 37 or 58 neurons, there was no difference between iPSCs and NPCs nor between the Days 37 and 58 neuronal samples. This suggests that between the NPC and the terminally differentiated neuron stage, there is a change in DNA methylation at the loci that constitute the clock, but these probes are not altered as cortical neurons age in culture. Previous studies of iPSC-derived neurons using the same (33Qn1) line have demonstrated that at Days 37 and 58, these cells are functionally mature (Telezhkin et al., 2016), which could be one reason that these “clock” probes are no longer altered.

One of the main objectives of our study was to identify an epigenetic trajectory signature of iPSC differentiation into cortical neurons. One interesting observation from this was that one of the Day 37 terminally differentiated neuron samples clustered more closely with the Day 58 terminally differentiated neuron samples than with other samples of the same cellular stage. We considered that this sample may have “aged” faster than the other Day 37 neurons. However, upon further investigation, this sample actually had the second lowest epigenetic age using the Steg clock and has the second highest age using the Horvath clock of the Day 37 group. This could therefore suggest that the probes used to determine epigenetic age are not contributing to the epigenetic trajectory signature we identified that distinguished between the different cellular stages.

Our pathway analysis highlighted that probes comprising the epigenetic trajectory signature are involved in “neuron projection morphogenesis,” “cell growth,” and “movement of cell or subcellular component.” The gene–gene interaction network analysis of the epigenetic trajectory signature identified a highly connected, epigenetically altered subnetwork of 60 genes, featuring 158 interactions. *STAT3* was the most connected gene in the SCC subnetwork. This gene is known to be involved in neuronal survival and function; for example, *STAT3* and other members of the JAK/STAT pathway have been shown to play key roles in the control of neuronal proliferation, survival, and differentiation (Yadav et al., 2005; Snyder et al., 2011). Primary neuronal and SH-SY5Y cells have been shown to be highly susceptible to treatment with the STAT3 inhibitor tryphostin, with a significant percentage of both cell types (80–100%) dying even at low concentrations (Yadav et al., 2005). The fact that *STAT3* was identified as a hub gene by the network analysis highlights its importance in the epigenetic trajectory signature, which is further confirmed by its already proven pivotal role in the development and differentiation of neurons.

To confirm that there were corresponding gene expression changes alongside methylation changes in our loci, we used the online tool created by Burke et al. (2020). This highlighted that 53 of our 60 most strongly connected genes (including *STAT3* and other epigenetic modulators) do undergo significant changes in both gene expression and methylation throughout overall neuronal differentiation. This suggests that the epigenetic changes occurring in the majority of genes in the SCC are having functional consequences in the differentiation of iPSCs into neurons.

There are some limitations to our study. First, our “oldest” time point was the Day 58 neurons, and it would be interesting to study neurons that have been cultured for longer periods to investigate whether these eventually develop a postnatal epigenetic age, and what changes occur to the epigenetic trajectory signature. Second, it would also be of interest to explore the epigenetic trajectory signature in inducible neurons (iNs), which are neurons generated directly from fibroblasts that do not go through the intermediate stem cell phase (Mertens et al., 2015). This is because previous studies have highlighted that iNs exhibit age-dependent nucleocytoplasmic compartmentalization and retain the age-related transcriptomic profiles and epigenetic age of their donor, which is lost in iPSC-derived neurons and so may be a more appropriate system for modeling age-related disease (Mertens et al., 2015). Third, we have only used one cell line (33Qn1) in our study, and it will be important for future studies to examine other cell lines, particularly ones harboring mutations known to be present in age-related neurological disease. In addition, the present study has utilized technical replicates, and future studies should characterize multiple iPSC clones for each line as the use of biological replicates would make the results more generalizable and robust. Fourth, future studies could also validate the epigenetic signature using an alternative technology such as bisulfite sequencing. Finally, in the current study, we have leveraged on a publicly available iPSC transcriptomics resource to explore patterns of gene expression through differentiation in various iPSC donors and clones over differentiation, highlighting differential expression of key genes we have identified in our epigenetic signature. However, it will be important in the future to explore whether the epigenetic patterns we identified lead to changes in gene expression in the same samples.

In conclusion, we have characterized genome-wide patterns of DNA methylation and identified an epigenetic trajectory signature comprising loci that become progressively hypermethylated or hypomethylated during the course of neuronal differentiation and maturation from iPSCs.

## Data Availability Statement

The dataset presented in this study can be found in an online repository. The raw and normalized DNA methylation data has been deposited in the NCBI Gene Expression Omnibus (GEO) under accession number GSE158089 and can be found here: https://www.ncbi.nlm.nih.gov/geo/query/acc.cgi?acc=GSE158089.

## Ethics Statement

The authors state that they have obtained appropriate institutional review board approval or have followed the principles outlined in the Declaration of Helsinki for all human or animal experimental investigations.

## Author Contributions

JI, JB, EC, KJ, and NA conducted laboratory experiments. JI, EP, MA, EG, and AJ undertook data analysis and bioinformatics. TK, AJ, NA, and KL conceived and supervised the project. JI, EP, and KL drafted the manuscript. All authors read and approved the final submission.

## Conflict of Interest

The authors declare that the research was conducted in the absence of any commercial or financial relationships that could be construed as a potential conflict of interest. The handling editor declared a shared affiliation with several of the authors JI, EP, TK, AJ, JB, KL, at time of review.

## Footnotes

1 http://stemcell.libd.org/scb/
