# Supplementary material for: Characterization of DNA Methylomic Signatures in Induced Pluripotent Stem Cells During Neuronal Differentiation
Source: Front Cell Dev Biol. 2021 Jul 1;9:647981. doi: 10.3389/fcell.2021.647981 (PMC8281298; doi:10.3389/fcell.2021.647981)

Supplementary Figure 1: Clustering of 14 samples based on Euclidean distance to prior Trajectory inference analysis. One sample from Day 37 is clustered together with the Day 58 samples.

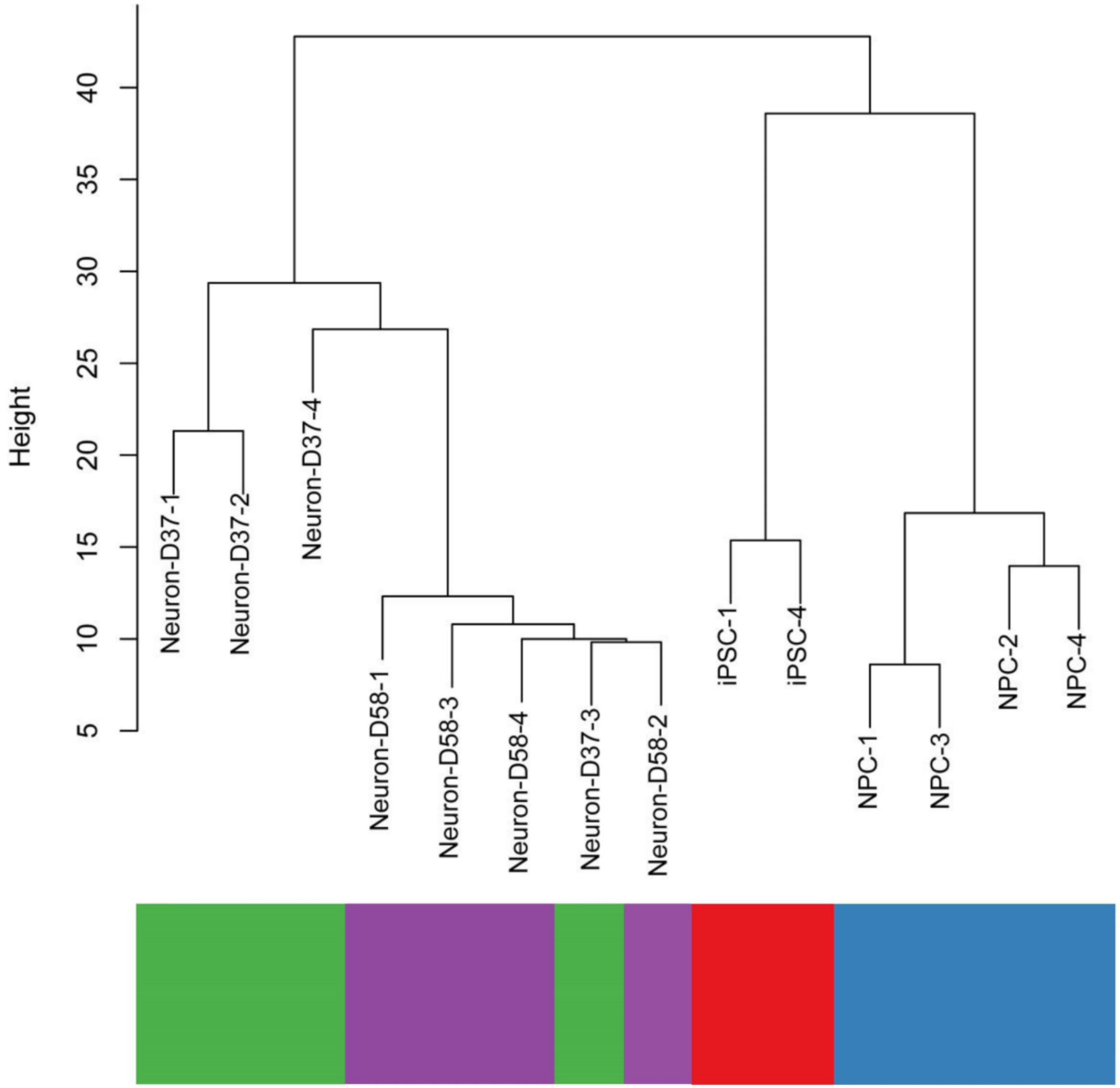

Supplementary Figure 2: Pathway analysis of the epigenetic trajectory signature. Gene ontology (GO) enrichment analysis was performed on the 6,843 loci that become progressively hypomethylated or hypermethylated throughout neuronal differentiation. Shown are the 18 false discovery rate (FDR) significant pathways. Pathways are ordered by their FDR P-value, which is illustrated using the color bar. Further details on pathways, including GO term, the number of genes in the pathway, genes present and exact P-values can be found in Supplementary Table 3.

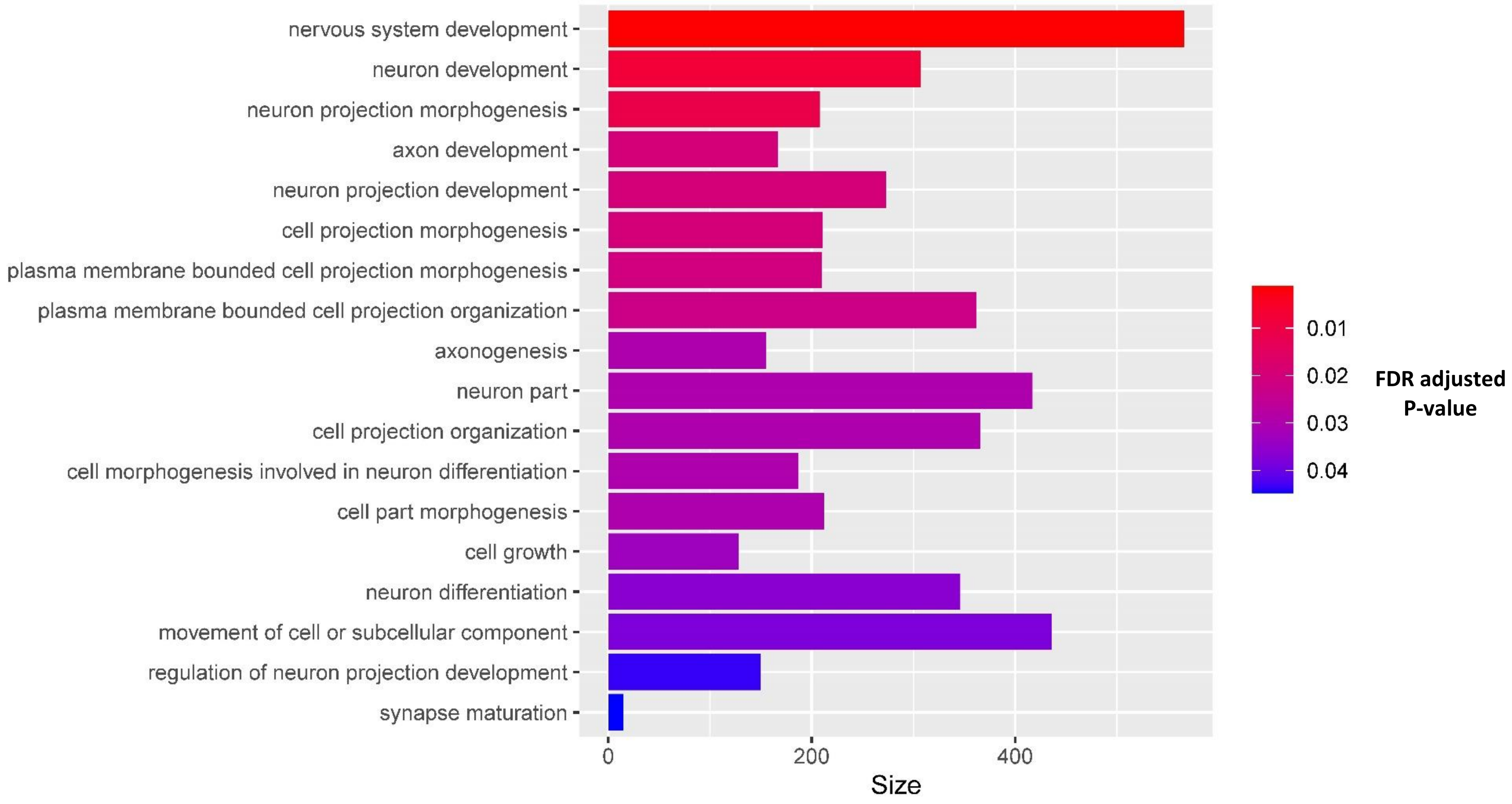

Supplementary Figure 3: Enrichment analysis of genomic features and regions annotated to the epigenetic trajectory signature. Shown are the percentage of probes annotated to each genomic region or features related to CG content for progressively hypomethylated loci (blue: N = 4,954), progressively hypermethylated loci (red: N = 1,889), all loci in the signature (orange: N = 6,843) and all of the variably methylated loci (grey: N = 41,851). Abbreviations: TSS1500 (1500bp from transcription start site), TSS200 (200bp from transcription start site), UTR (untranslated region), island (CpG island), shelf (CpG island shelf), shore (CpG island shore). Further information on these enrichments, such as odds ratio (OR) and P-values can be found in Supplementary Table 4.

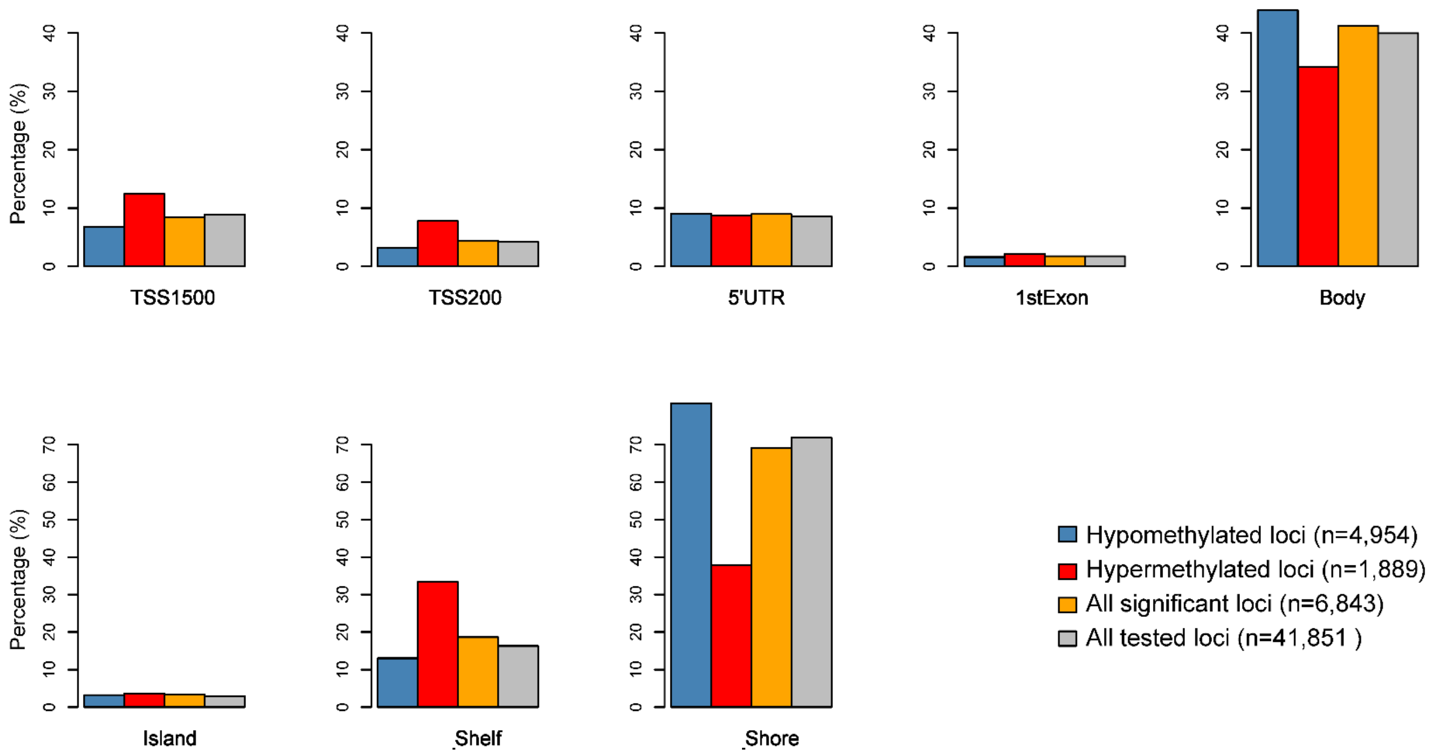

Supplementary Figure 4: Gene expression levels of our top four most connected genes throughout neuronal differentiation in the gene-gene interaction network analysis. Using the LIBD stem cell browser, created by Burke and colleagues (Burke *et al*, 2020), we visualized the gene expression changes throughout neuronal differentiation for our four most connected genes: **A)** *STAT3*, **B)** *ETS1*, **C)** *CD44* and **D)** *EGFR*. The square points at days 2, 4 and 6 in vitro (DIV) are the self-renewal condition, the circular points at 2, 4, 6 and 9 DIV are the accelerated dorsal condition, the circular points at 15 DIV are the NPCs, the circular points at 21 DIV are the rosettes, the circular points at 49, 63 and 77 DIV are neurons grown with rat astrocytes and the diamond points at 77 DIV are the pure neuronal condition. Each graph has the f-statistic and q-value statistic for the differential expression over the entirety of differentiation generated from the browser. For more information, the LIBD stem cell browser is available at http://stemcell.libd.org/scb/.

A)

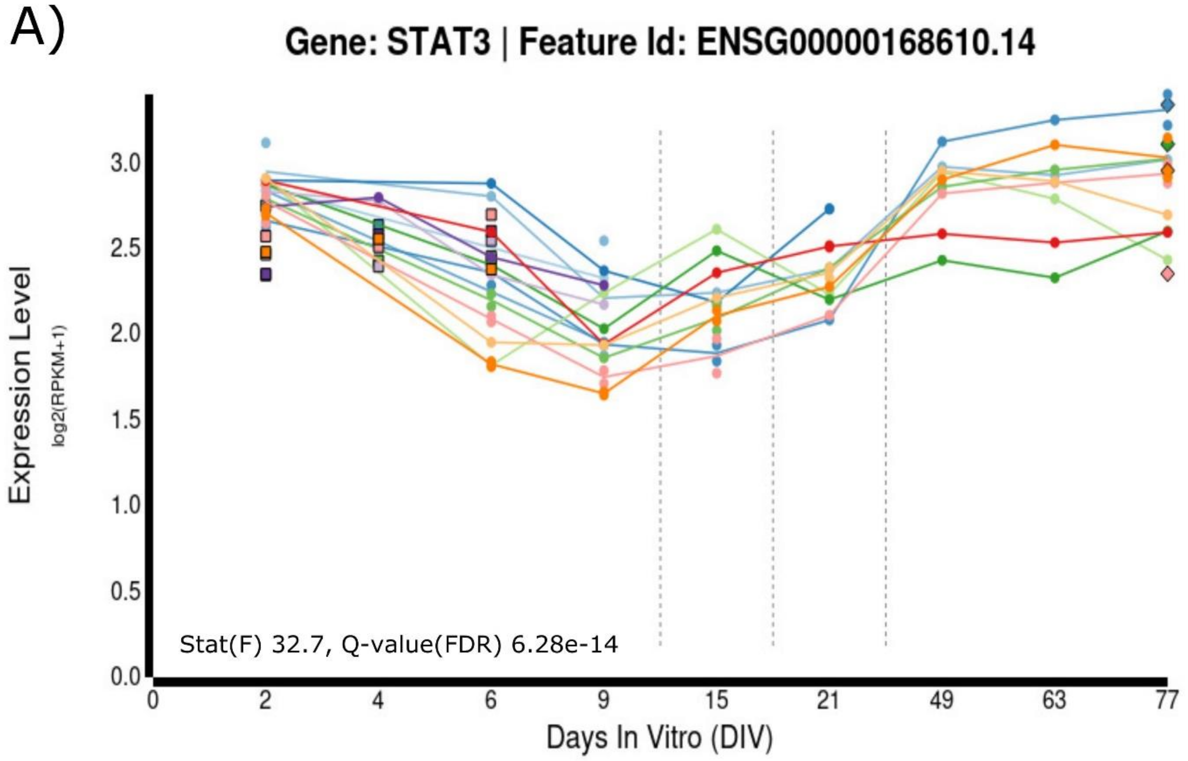

B)

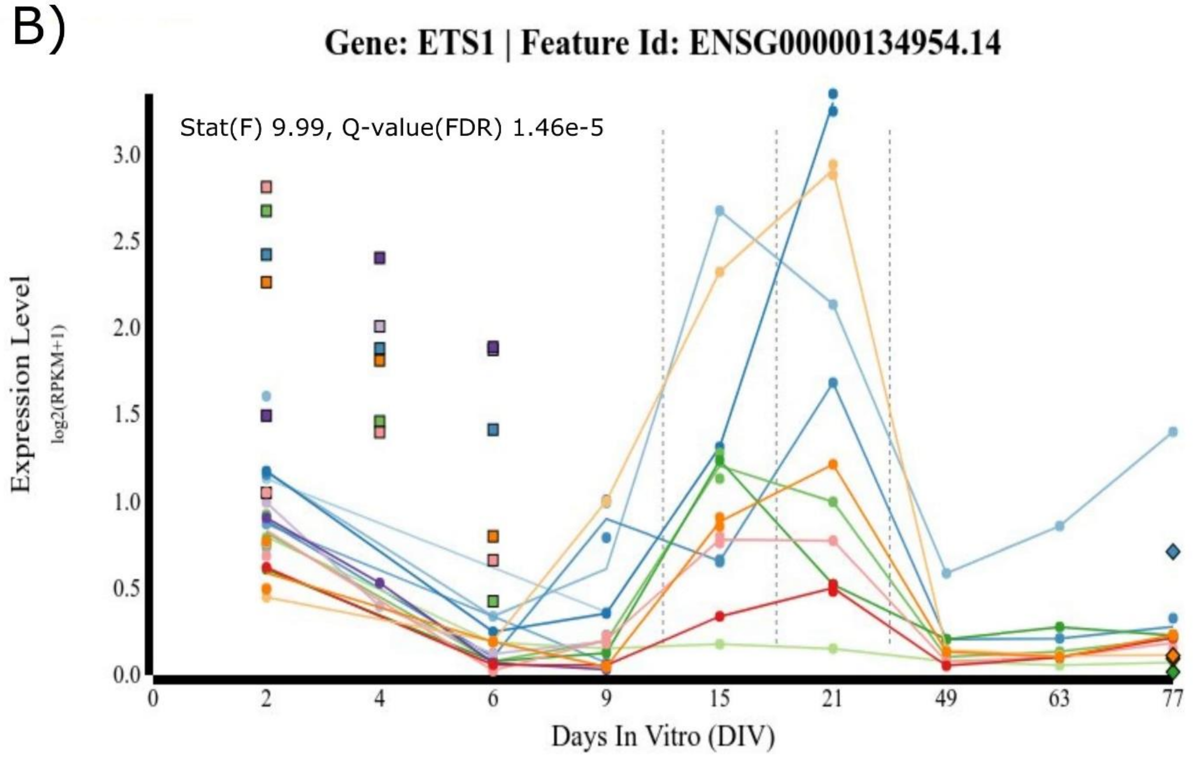

C)

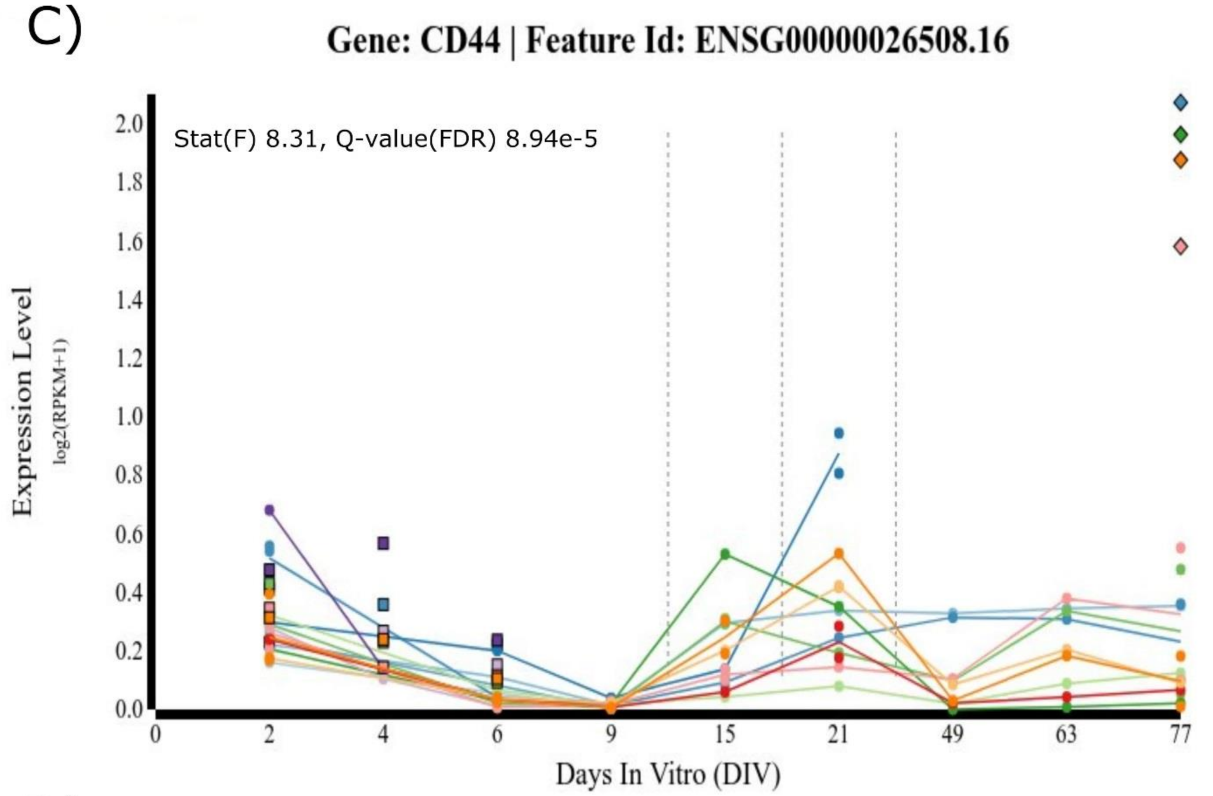
Gene: CD44 | Feature Id: ENSG00000026508.16
Stat(F) 8.31, Q-value(FDR) 8.94e-5
Expression Level
log2(RPKM+1)
Days In Vitro (DIV)

D)

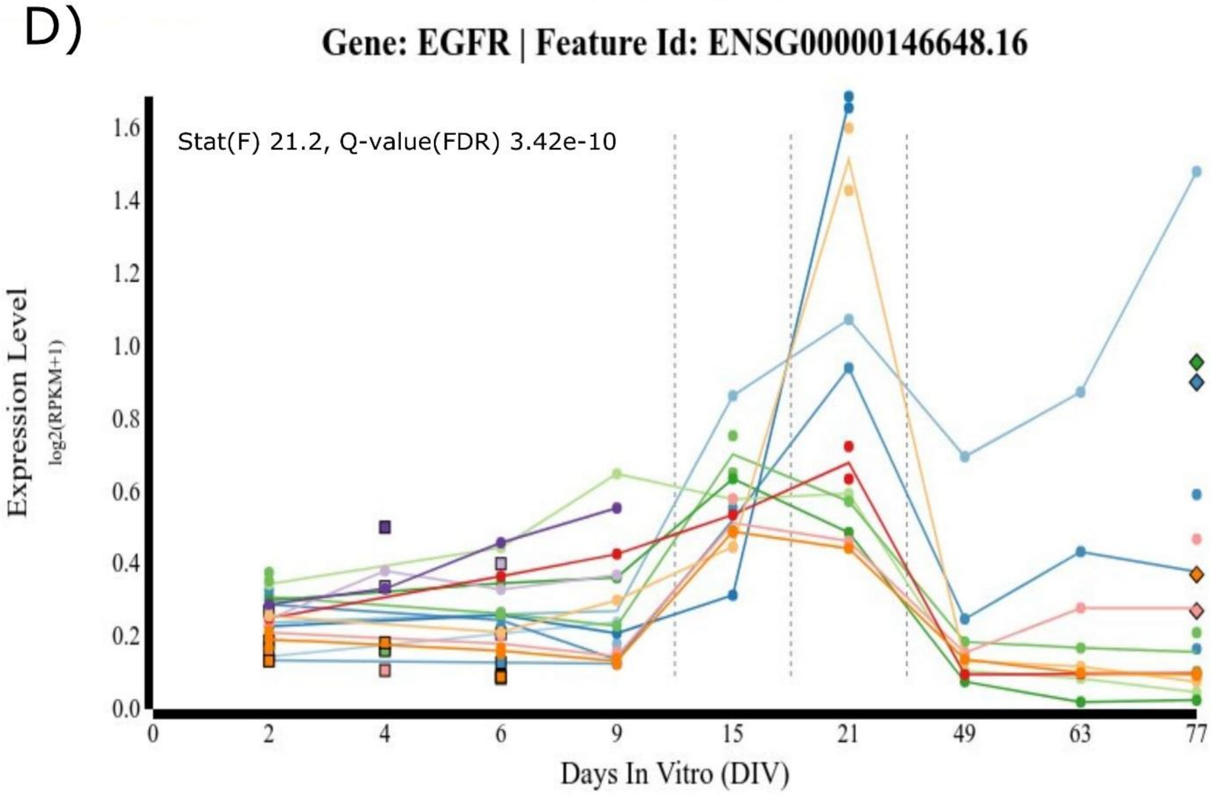
Gene: EGFR | Feature Id: ENSG00000146648.16
Stat(F) 21.2, Q-value(FDR) 3.42e-10
Expression Level
log2(RPKM+1)
Days In Vitro (DIV)

Supplementary Figure 5: Gene expression changes throughout neuronal differentiation for the most connected epigenetic modulators featured in our gene-gene interaction network analysis. Using the LIBD stem cell browser, created by Burke and colleagues (Burke *et al*, 2020), we visualized the gene expression changes throughout neuronal differentiation for two epigenetic modulators that featured in our gene-gene interaction network analysis: **A)** *DNMT3B* and **B)** *DNMT3A*. The square points at days 2, 4 and 6 in vitro (DIV) are the self-renewal condition, the circular points at 2, 4, 6 and 9 DIV are the accelerated dorsal condition, the circular points at 15 DIV are the NPCs, the circular points at 21 DIV are the rosettes, the circular points at 49, 63 and 77 DIV are neurons grown with rat astrocytes and the diamond points at 77 DIV are the pure neuronal condition. Each graph has the f-statistic and q-value statistic for the differential expression over the entirety of differentiation. For more information, the LIBD stem cell browser is available at http://stemcell.libd.org/scb/.

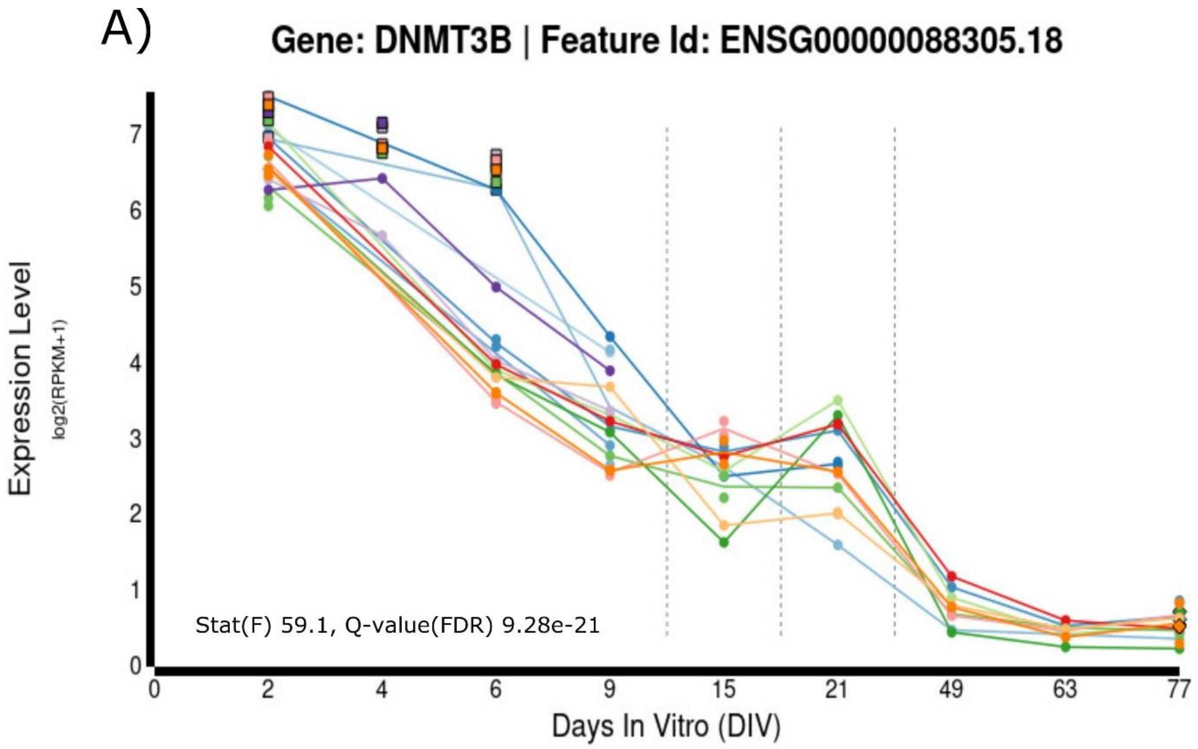

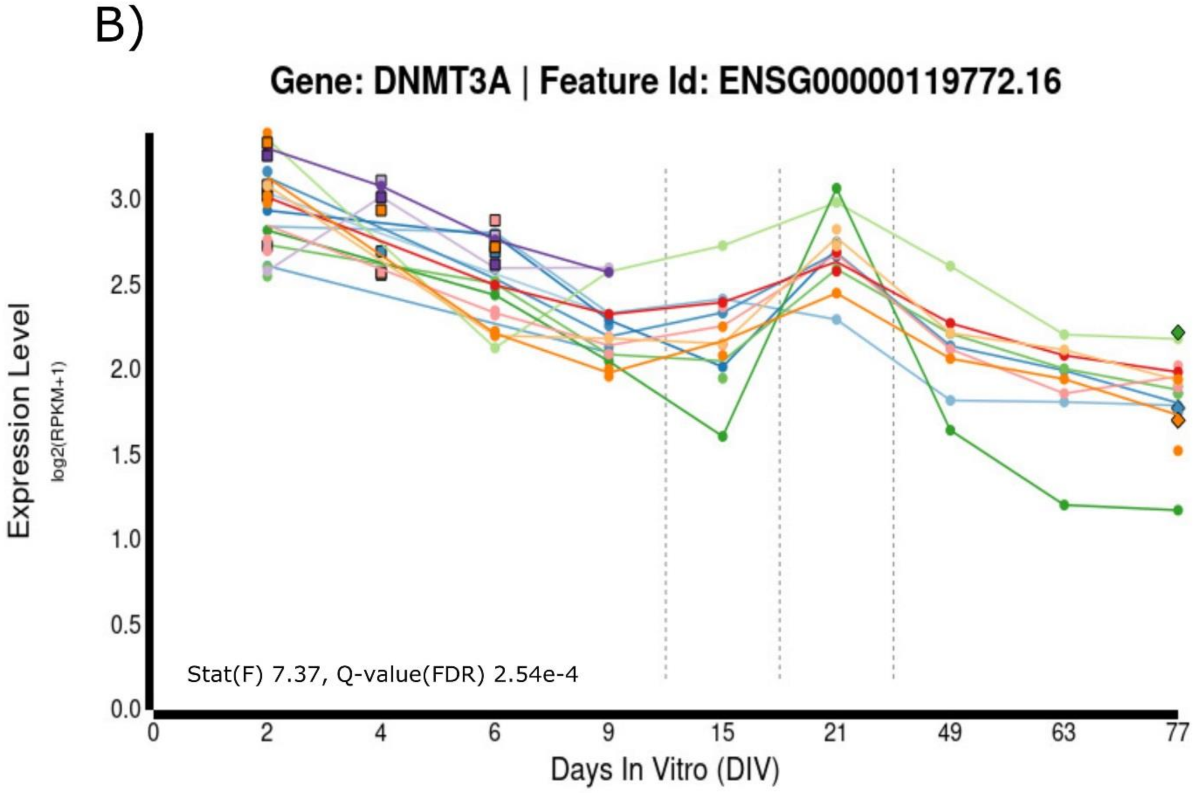

Supplement: Supplementary file 2 [file Data_Sheet_1.PDF]
